# Intestinal vitamin D receptor knockout protects from oxazolone-induced colitis

**DOI:** 10.1038/s41419-020-2653-3

**Published:** 2020-06-15

**Authors:** Yongyan Shi, Ziyun Liu, Xuewei Cui, Qun Zhao, Tianjing Liu

**Affiliations:** 1 Department of Pediatrics, Shengjing Hospital of China Medical University, Shenyang, China; 2 Department of Medicine, Division of Biological Sciences, University of Chicago, Chicago, USA; 3 Department of Pediatric Orthopedics, Shengjing Hospital of China Medical University, Shenyang, China

**Keywords:** Inflammatory bowel disease, Experimental models of disease

## Abstract

Crohn’s disease (CD) and ulcerative colitis (UC) actually had different pathological mechanisms, as the former was mainly induced by Th1 and Th17 response and the latter by Th2 response. Our previous study found that oxazolone-induced Th2-mediated colitis could not be attenuated by vitamin D supplementation. This study investigated the influence of intestinal vitamin D receptor (VDR) knockout on oxazolone-induced colitis and explored the possible immunological mechanism. Intestinal VDR knockout mice had milder oxazolone-induced colitis than wildtype controls, as demonstrated by less body weight decrease and faster recovery, more intact local structure, reduced cell apoptosis, and better preserved barrier function. Th2-mediated inflammation was significantly inhibited by VDR deficiency. Meanwhile, the percentage of invariant natural killer T (iNKT) cells did not increase as much in intestinal VDR knockout mice as in wild-type controls, nor did the iNKT cells develop normally as in the controls. Intestinal VDR knockout protected against oxazolone-induced colitis in mice by blocking Th2 cell response and reducing the function of intestinal iNKT cells. Vitamin D status had no influence on the severity of colitis. This study may explain the diverse outcomes after vitamin D supplementation in literature and add some clue to the targeted therapy of IBD.

## Introduction

Vitamin D deficiency had been closely associated with auto-immunological diseases including IBD. Clinical trials on vitamin D deficiency and IBD were yielding contradictory results concerning the development, disease activity and remission rate^1,2^. Meanwhile, the effect of vitamin D supplementation in treating IBD was also controversial^3,4^. The controversy might root in the different mechanisms between Crohn’s disease (CD) and ulcerative colitis (UC), or even the diverse immunological sub-types of the two main categories. As previously reported UC and CD shared 30% identical genetic risk coli and were quite different in the site of involvement, clinical manifestations and immunological process^5^.

Based on the diverse pathogenesis of IBD, several murine models had been developed. Dextran sulfate sodium (DSS) and 2,4,6-trinitrobenzenesulfonic acid solution (TNBS) were frequently used to induce Th1/Th17-associated colitis that represented the Crohn’s disease, while oxazolone was used to simulate ulcerative colitis by inducing Th2 inflammatory response. Immunological defect models, such as IL-10 knockout and sensitized Rag models had spontaneous colitis due to complicated imuno-pathological mechanisms^6^. The role of vitamin D and its receptor VDR had been tested in most of the models. In IL-10 knockout mice and DSS-induced colitis models, VDR exhibited protective effect on intestinal structure and barrier function^7,8^. Global VDR knockout mice had much more severe TNBS-induced colitis compared to their wild-type littermates. While in those VDR global knockout mice with intestinal-specific human VDR knock-in, the colitis would be significantly inhibited^9^. This was probably due to the suppressive effect of vitamin D signaling pathway on Th1 andTh17 cell response.

However, the role of vitamin D and VDR in oxazolone-induced colitis had not been fully elucidated. Our previous research compared the effect of vitamin D supplementation on TNBS and oxazolone-induced colitis. We found that unlike TNBS-induced colitis, oxazolone-induced colitis would not be alleviated by paricalcitol, a bio-active vitamin D analog^10^. This was probably due to the simultaneous activation of Th2 and Treg response by vitamin D that compromised each other. However, the role of vitamin D receptor was not specified in that study. VDR itself was an indispensable factor to the development and maturation of intestinal immune cells. Intestinal VDR knockout may result in autophagy deficiency, decrease in the number and function of Paneth cells and dysbiosis^11^. Besides, VDR deficient gut had less and underdeveloped invariant nature killer T (iNKT) cells as well as significantly reduced CD8*αα*T cells^12^.

In this study, oxazolone would be used to induce colitis in intestinal-specific VDR knockout mice. Changes in general manifestation, local structure, barrier function, and immunological response would be observed and analyzed. We mainly focused on the regulatory role of VDR on Th2-mediated inflammatory response and function of iNKT cells. Meanwhile, the influence of serum vitamin D status was also observed in order to specify the role of VDR.

## Results

**Fig. 1:**
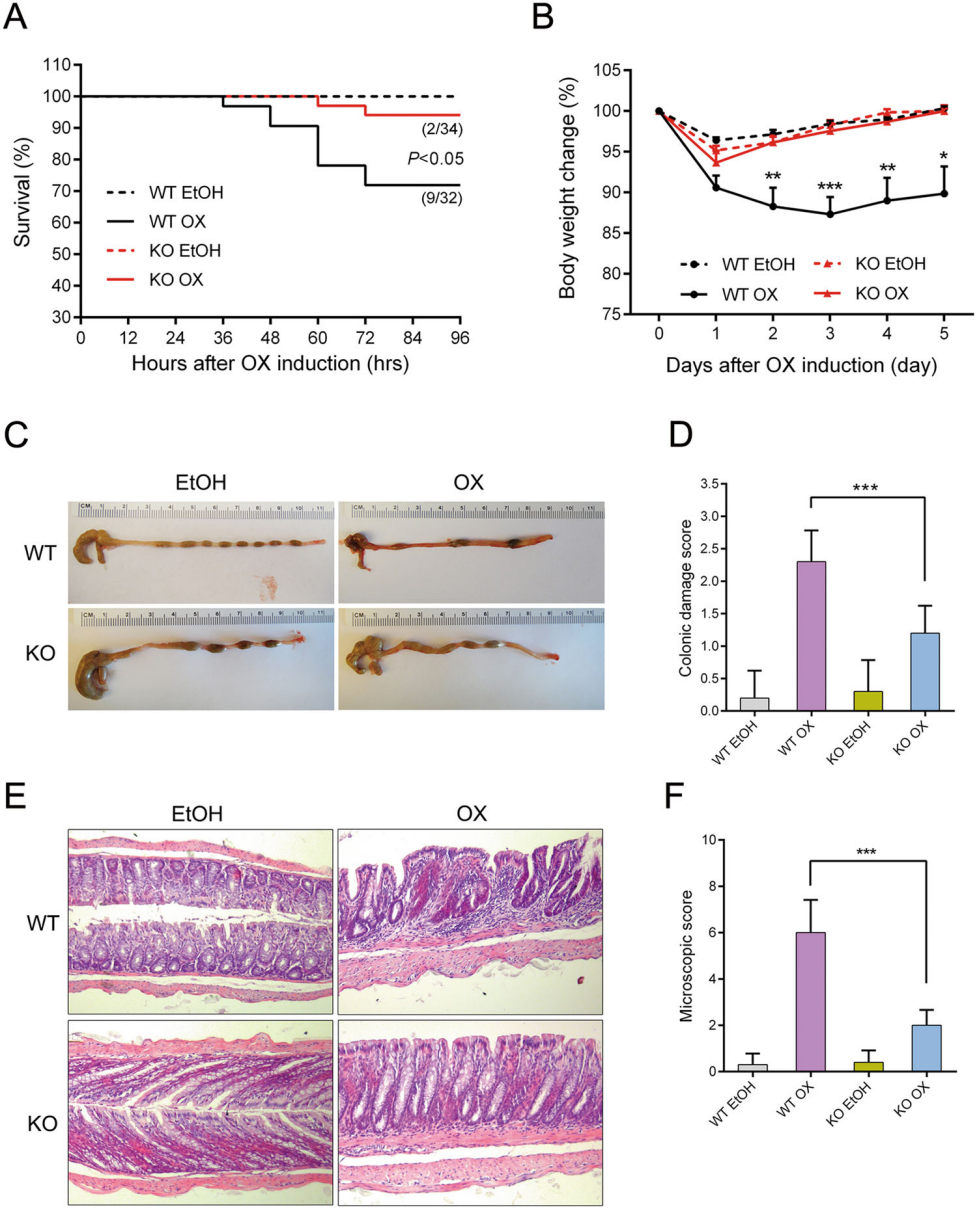
Presentations of wild-type and intestinal VDR knockout mice after oxazolone challenge. **a** Survival rate (percentage of the original number) (9/32 died in the WT OX group and 2/34 died in the KO OX group). **b** Body weight changes (percentage of the original body weight) over time (days) in theWT and KO groups following 50% ethanol or oxazolone treatment. The *P* value reflected the comparison between WT OX and KO OX group. *P < 0.05, **P < 0.01, ***P < 0.001. (*n* = 8 in each group). **c** Typical gross morphology of the colon on day 5 from each group. **d** Colonic damage scores of each group. *** *P* < 0.001 (*n* = 10 in each group). **e** Haematoxylin and eosin staining of colons on day 5 following different treatment methods. **f** Microscopic scoring of each colonic slide based on haematoxylin and eosin staining. Original magnification: 200×. ***P < 0.001 (*n* = 10 in each group). WT wild-type mice, KO intestinal-specific VDR knockout mice, EtOH 50% ethanol, OX oxazolone, WT EtOH wild-type mice treated with 50% ethanol, KO EtOH intestinal-specific VDR knockout mice treated with 50% ethanol, WT OX wild-type mice treated with oxazolone, KO OX intestinal-specific VDR knockout mice treated with oxazolone.

**Fig. 2:**
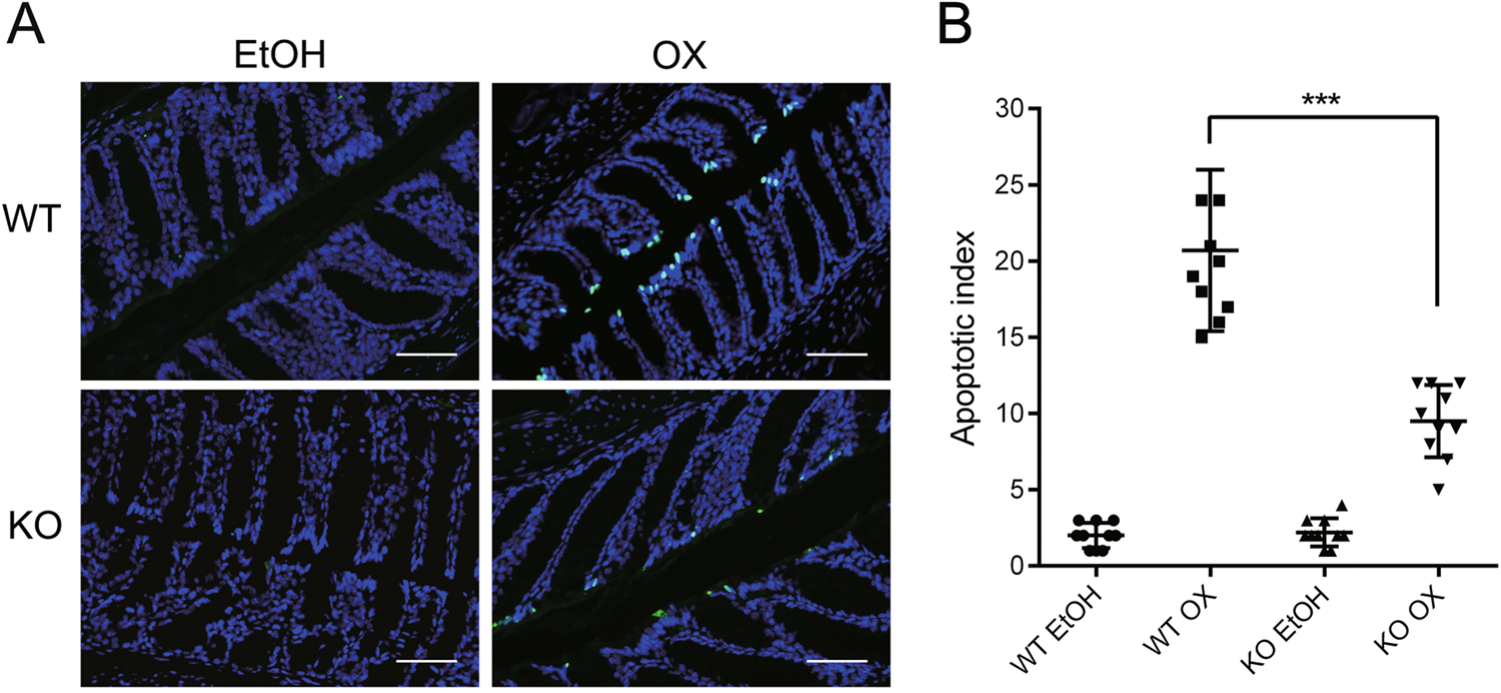
Intestinal VDR knockout ameliorated epithelial cell death in oxazolone-induced colitis. **a** Representative TUNEL staining of each treatment group on day 2. Green spots indicate TUNEL-positive apoptotic cells, Original magnification: 200×, bar = 100 μm. **b** Apoptotic index in each group. The apoptotic index was defined as the percentage of TUNEL-positive crypts in 100 randomly chosen crypts in each colon slide. ***P < 0.001 (*n* = 10 in each group). WT wild-type mice, KO intestinal-specific VDR knockout mice, EtOH 50% ethanol, OX oxazolone, WT EtOH wild-type mice treated with 50% ethanol, KO EtOH intestinal-specific VDR knockout mice treated with 50% ethanol, WT OX wild-type mice treated with oxazolone, KO OX intestinal-specific VDR knockout mice treated with oxazolone.

Wild-type mice challenged by oxazolone (WT OX) presented with significant body weight loss in the first three days and 9/32 mice died. The body weight started to recover since the fourth day. Intestinal vitamin D knockout challenged by oxazolone (KO OX) showed very mild colitis changes. Their body weight loss and recovery time were very close to those that were given the dissolvent only. The intestinal structure disruption and infiltration of the inflammatory cells was the most severe in WT OX, while that of KO OX was very close to normal. The macroscopic and microscopic scores of KO OX were significantly lower than those of WT OX (Fig. 1). TUNEL staining showed obviously increased epithelial cell apoptosis in WT OX. Although apoptosis did increase in KO OX, it was much less obvious compared to WT OX (Fig. 2).

**Fig. 3:**
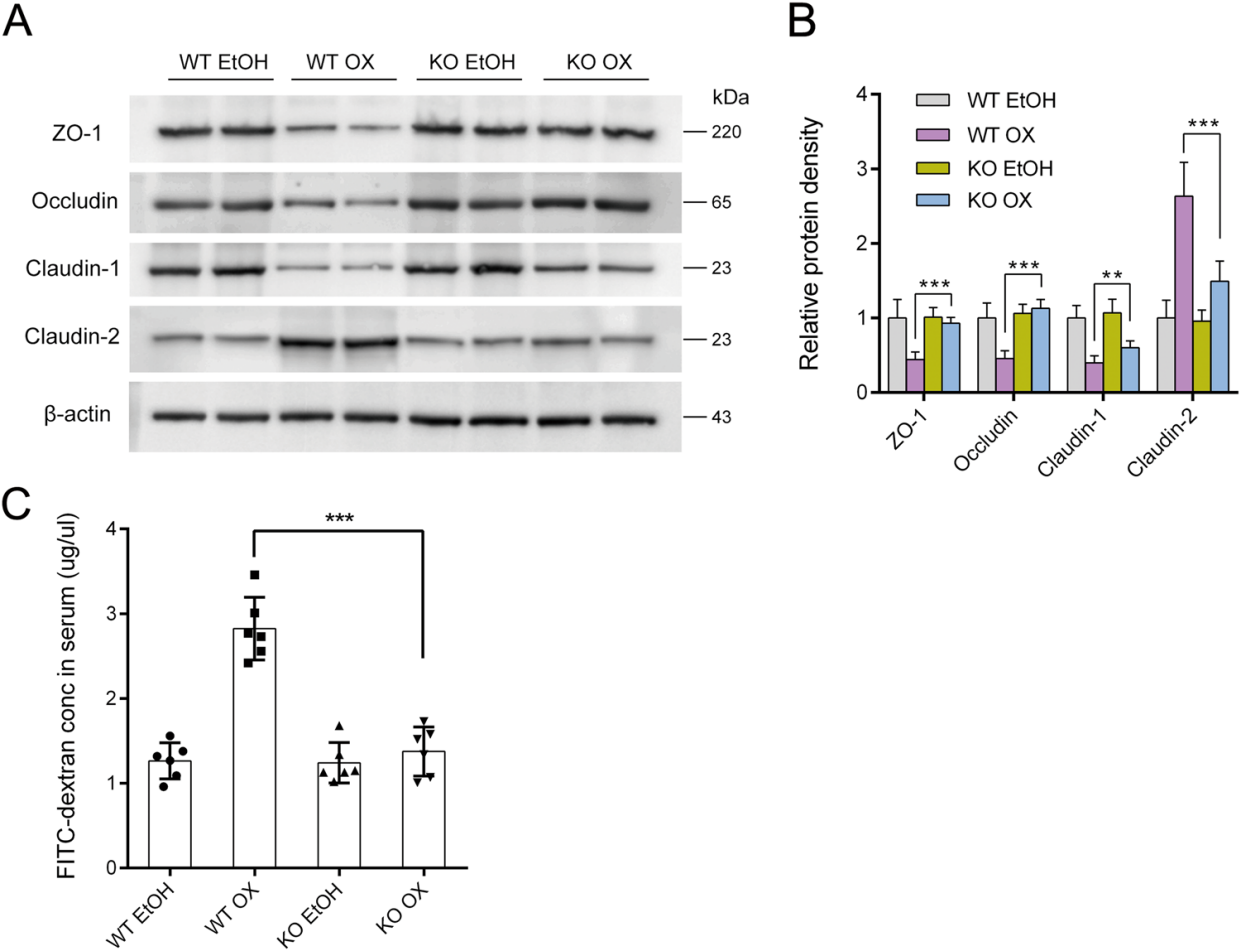
Intestinal VDR knockout preserved intestinal epithelial barrier by regulating the expressions of tight junction proteins. **a** Western blotting **b** Relative density comparisons of the colonic mucosal levels of tight junction proteins in different treatment groups on day 2. (*n* = 6 in each group). **c** Intestinal permeability to 4-kDa FITC-dextran was measured at 3 h after gavage (*n* = 6 in each group). The *P* value referred to the comparison between WT OX and KO OX group. WT EtOH wild-type mice treated with 50% ethanol, KO EtOH intestinal-specific VDR knockout mice treated with 50% ethanol, WT OX wild-type mice treated with oxazolone, KO OX intestinal-specific VDR knockout mice treated with oxazolone. **P < 0.01, ***P < 0.001.

Then we looked at the influence of oxazolone on barrier function. Oxazolone caused significant decrease in the expression of tight junction proteins like ZO-1, occludin, and claudin-1, while significant increase in the expression of pore-forming protein claudin-2. These changes were hardly seen in KO OX, as their expressions were quite close to those challenged with the dissolvent only. Serum FITC-dextran concentration was much lower in KO OX than in WT OX, indicating better preserved barrier function in KO OX that was almost the same as normal (Fig. 3).

**Fig. 4:**
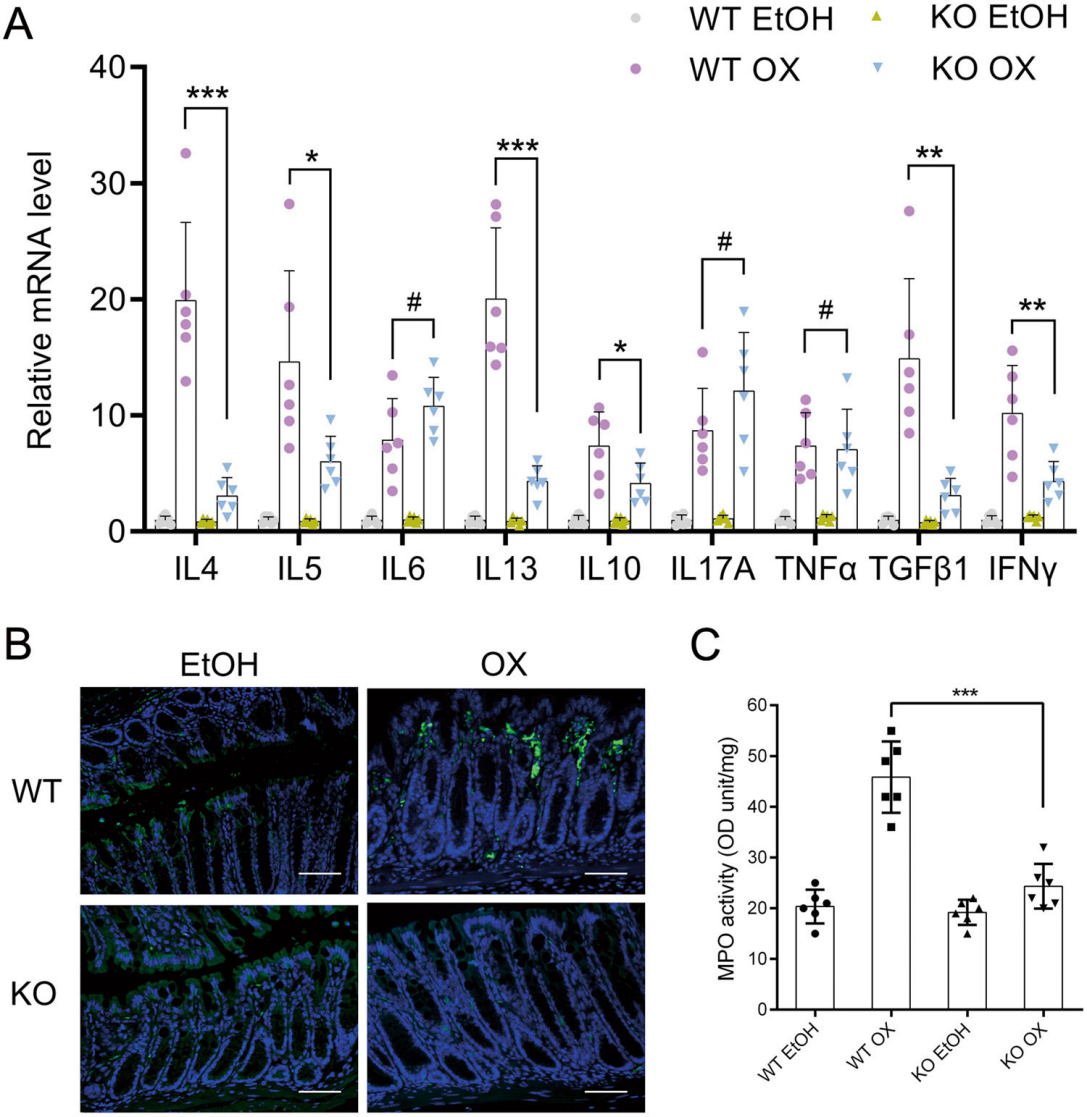
Intestinal VDR knockout mice had suppressed Th2 cytokine expressions and less infiltration of inflammatory cells. **a** Relative mRNA expression of cytokines in different treatment groups on day 2 by real-time PCR. **b** Immunofluorescence staining of colons with anti-CD4 antibody on day 2 after different treatments. Original magnification: 200×, bar = 100 μm. **c** Myeloperoxidase (MPO) activity. WT wild-type mice, KO intestinal-specific VDR knockout mice, EtOH 50% ethanol, OX oxazolone, WT EtOH wild-type mice treated with 50% ethanol, KO EtOH intestinal-specific VDR knockout mice treated with 50% ethanol, WT OX wild-type mice treated with oxazolone, KO OX intestinal-specific VDR knockout mice treated with oxazolone. #*P* > 0.05, *P < 0.05, **P < 0.01, ***P < 0.001 (*n* = 6 in each group).

Real-time PCR was performed to analyze the levels of cytokines. Dissolvent did not cause any difference in cytokine levels between the WT and OX group. After oxazolone challenge, inflammatory cytokines generally increased in both groups. TNF*α* and IL-17 increased similarly. Th1-related cytokines increased more in KO than in WT. Th2-related cytokines, including IL-4, IL-5, and IL-13, as well as Treg-related IL-10, increased much less in KO than in WT group. This showed slightly induced Th1-response but greatly reduced Th2-response in KO OX. As a result, less inflammatory cells trafficked to the intestine in KO OX (Fig. 4).

**Fig. 5:**
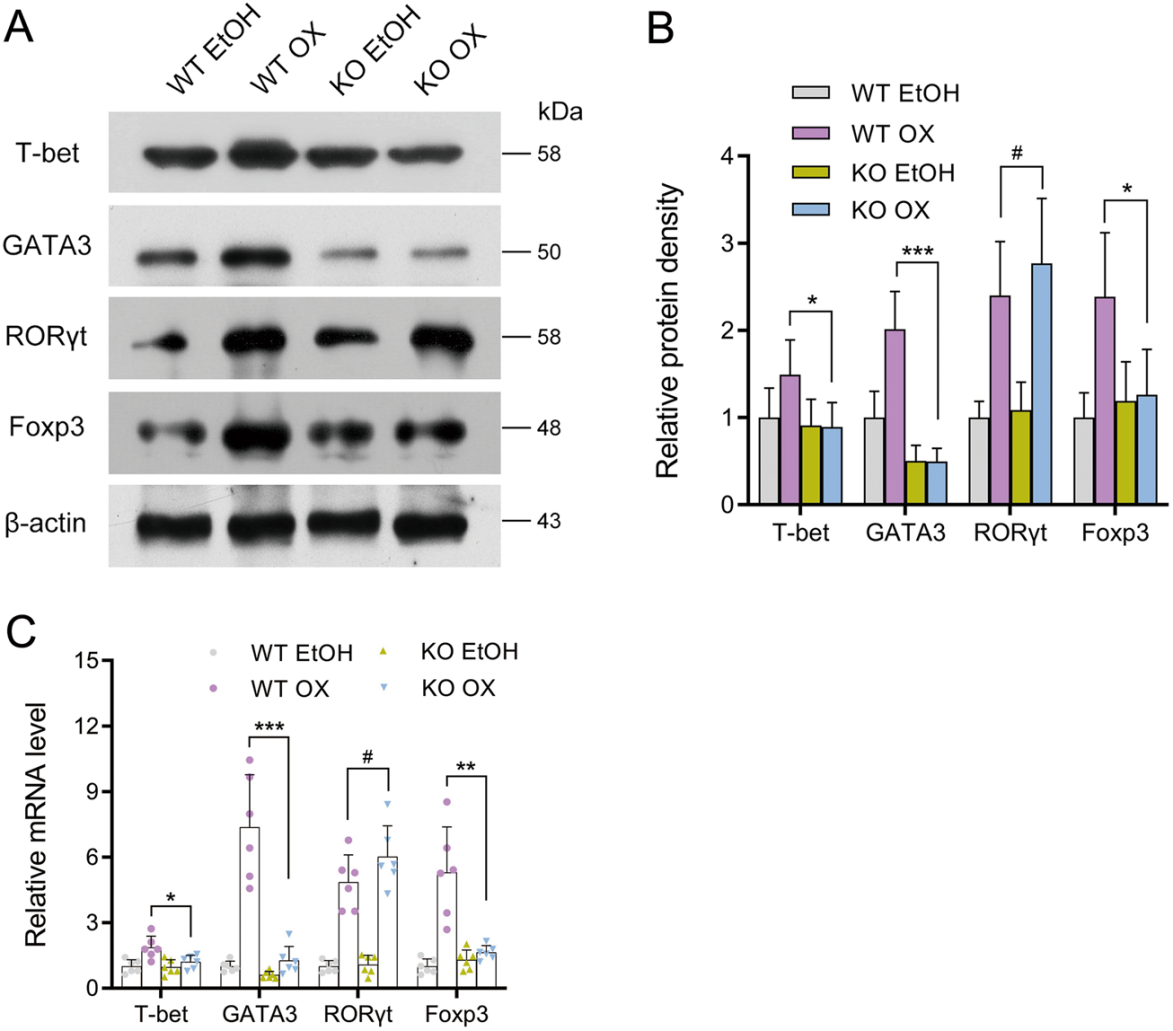
Factor of Th2 response decreased in expression after oxazolone stimulation in intestinal VDR knockout mice compared to controls. **a** Western blotting **b** Relative density comparisons of representative factors of Th1, Th2, Th17, and Treg cells. **c** Relative mRNA expression of the transcriptional factors in different treatment groups on day 2 by real-time PCR. WT EtOH: wild-type mice treated with 50% ethanol. KO EtOH intestinal-specific VDR knockout mice treated with 50% ethanol, WT OX wild-type mice treated with oxazolone, KO OX intestinal-specific VDR knockout mice treated with oxazolone. #*P* > 0.05, *P < 0.05, **P < 0.01, ***P < 0.001. (*n* = 6 in each group).

**Fig. 6:**
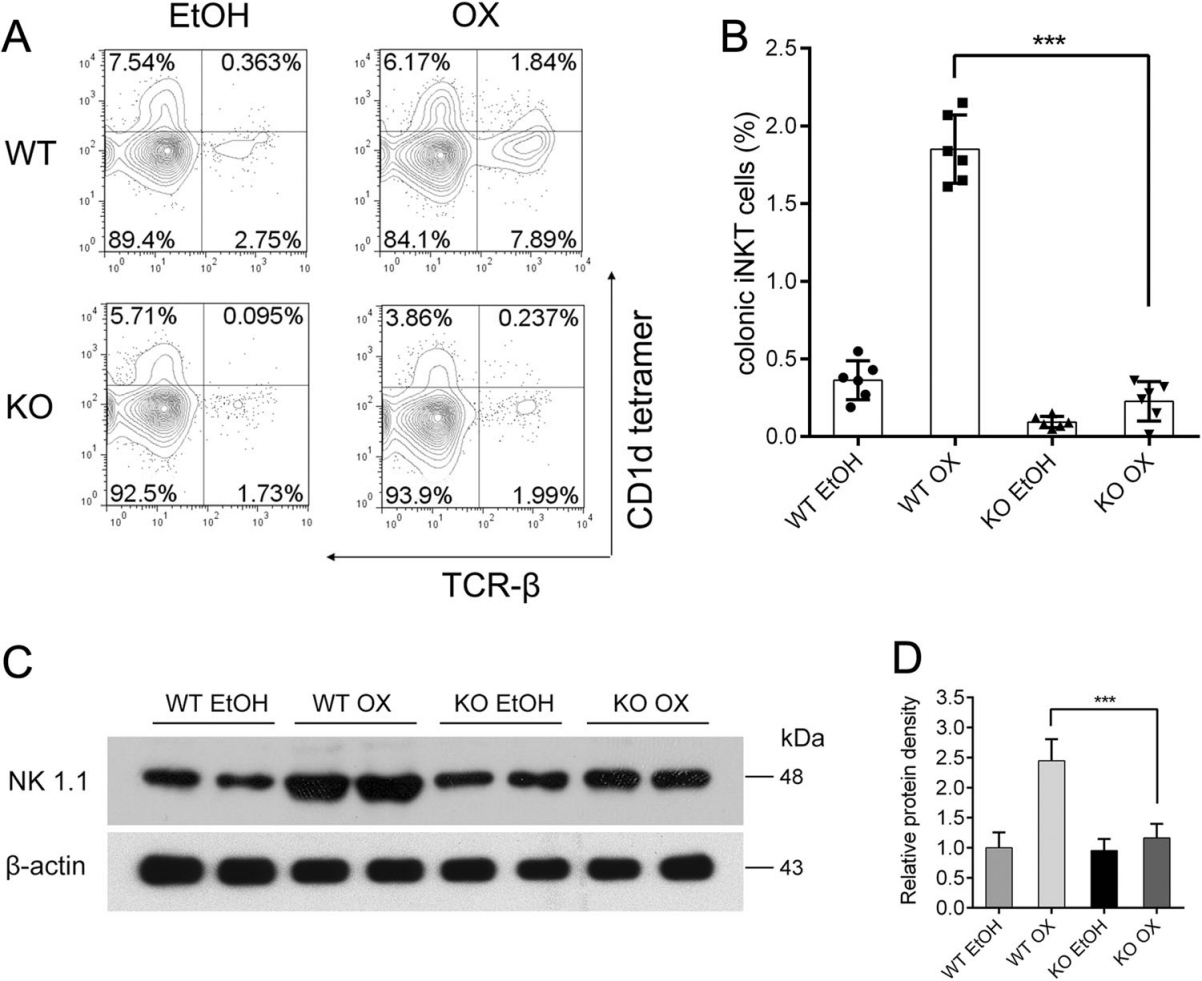
Decreased frequency of colonic iNKT cells and less maturity after oxazolone challenge in intestinal VDR knockout mice compared to controls. The recruitment of iNKT cells in lamina propria extracted from the colon of mice in each group were examined by flow cytometry. **a** The frequency of iNKT cells. **b** The percentage of iNKT cells in each group. **c** Western blotting. **d** Relative density comparisons of NK1.1 expression. WT wild-type mice, KO intestinal-specific VDR knockout mice, EtOH 50% ethanol, OX oxazolone, WT EtOH wild-type mice treated with 50% ethanol, KO EtOH intestinal-specific VDR knockout mice treated with 50% ethanol, WT OX wild-type mice treated with oxazolone, KO OX intestinal-specific VDR knockout mice treated with oxazolone. ***P < 0.001 (*n* = 6 in each group).

Then we investigated the transcriptional factors of T cells. Th2-specific factor, GATA3, expressed less in KO than in WT. After oxazolone challenge, GATA3 in WT rose dramatically in accordance with the Th2 nature of oxazolone-induced colitis. However, it did not change in KO, meaning that Th2 response was hardly initiated in KO. The expression of T-bet and Foxp3 was also less in the KO group but the difference was not as obvious as in GATA3. ROR*γ*t, the factor of Th17, slightly increased. These findings confirmed that the Th2 response was highly suppressed in KO OX, while the Th1 and Treg were slightly suppressed (Fig. 5). Flow cytometry found that iNKT cells that expressed both CD1d and TCR-*β* could be stimulated by OX in WT and increase significantly in percentage, but this increase was quite minor in KO. NK1.1, a marker of mature iNKT cells, also increased little in expression in KO mice after oxazolone challenge compared to the dramatic increase in normal controls. Failure of iNKT activation and maturation also contributed to the lack of inflammatory response in KO after oxazolone challenge (Fig. 6).

**Fig. 7:**
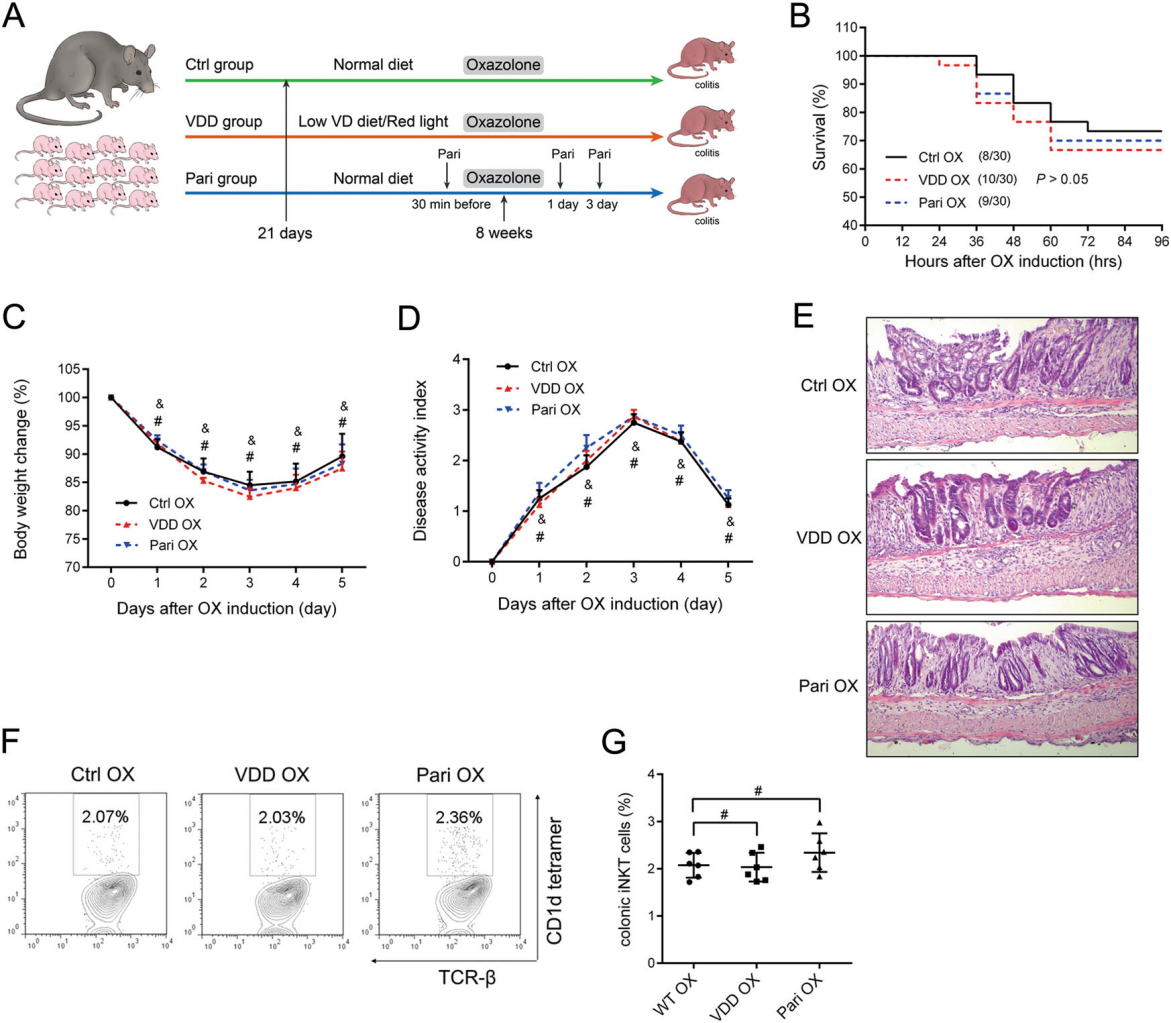
Vitamin D status in wildtype mice did not have any influence on disease severity of oxazolone-induced colitis. **a** The study regimen. **b** Survival rates (8/30 died in the Ctrl OX group, 9/30 in the VDD OX group and 10/30 in the Pari OX group). *#P* > 0.05. **c** Body weight change (*n* = 6 in each group) **d** Disease activity scores (*n* = 8 in each group). *#P* > 0.05 representing the comparison between Ctrl OX and VDD OX. *&P* > 0.05 representing the comparison between Ctrl OX and Pari OX. **e** Representative haematoxylin and eosin staining of colons on day 5 in each group. Original magnification: 200×. **f** The frequency of iNKT cells. **g** The percentage of iNKT cells in each treatment group. #*P* > 0.05. Ctrl OX wild-type mice receiving normal diet and light before and during treatment with oxazolone, VDD OX wild-type mice that were vitamin D deficient before and during treatment with oxazolone, Pari OX wild-type mice that were given paricalcitol before and during treatment with oxazolone.

In order to further clarify, which member of the vitamin D/VDR pathway was actually causing those changes, we tested the effect of vitamin D status on oxazolone-induced colitis in WT mice. We found that neither vitamin D deficiency nor vitamin D supplement had any influence on the survival rate, body weight change, disease activity, and histological structure after oxazolone-induction in WT mice. The percentage of iNKT cells was similar in three groups. This finding confirmed that VDR, rather than the vitamin D level, actually functioned in the suppression of oxazolone-induced colitis (Fig. 7).

## Discussion

Oxazolone-induced colitis was a typical Th2-mediated colitis that was close to the pathogenesis of UC. Oxazolone stimulated the response of Th2 and iNKT cells, leading to a characteristic elevation of IL-13, as well as other cytokines including IL-4 and IL-5^13^. The elevation of IL-4 in turn further stimulated the Th2 cells to produce more IL-4, and thus aggravated the inflammatory response in a positive feedback manner^14^. Body weight loss, intestinal structure damage, barrier dysfunction, and increased epithelial cell death took place soon after the administration. Those that survived the first several days would gradually recover around the fifth day, as was observed in our and other studies^15^.

Based on the immunological characters of oxazolone-induced colitis, many targets had been tested as to inhibit or alleviate the inflammatory response. When STAT6, a downstream factor of IL-13, was blocked, the pore-forming protein Claudin-2 will be decreased and the colitis could be largely alleviated^16^. Simultaneous inhibition of IL-4 and IL-13 had been reported to have better outcome than IL-13 inhibition alone as in some cases IL-13 was not the only key initiator of the inflammation^17^. Our previous study tested the effect of vitamin D, a pronounced IBD protector, but it turned out that vitamin D did not show any protective effect on oxazolone-induced colitis^10^.

In this study, we induced colitis in an intestinal-specific VDR knockout model in order to further clarify the role of intestinal vitamin D signaling pathway in oxazolone-induced colitis. Intestinal VDR knockout could put the gut in a relatively normal background and eliminate the influence of systematic disturbance caused by global VDR dysfunction. Intestinal VDR^−/−^ mice had less body weight loss, higher rate of survival, and faster recovery compared to the WT controls. The macroscopic and microscopic structure of the colon was better preserved, the epithelial cell apoptosis was largely reduced and the barrier integrity was significantly maintained in the intestinal VDR^−/−^ mice. These demonstrated that VDR removal might be of some protective effect on oxazolone-induced Th2-mediated colitis.

Traditionally, the vitamin D/VDR pathway had been acknowledged as a stimulator of Th2-mediated inflammation. Vitamin D could enhance the function of CD4^+^Th2 cells and promote the shift from Th1 to Th2-cell response in vitro^18^. VDR^−/−^ T cells had decreased Th2 function and produced less IL-4 than the WT counterparts^19^. In this study, the expression of IL-4, IL-5, and IL-13 increased much less in VDR^−/−^ mice than in the WT mice, demonstrating the dysfunction of Th2-cell response. Consequently, the downstream inflammatory response could not be adequately activated, so that the attack of inflammatory cells and cytokines was decreased and the structure and function of the intestinal epithelial cells was maintained. Similarly, VDR knockout would inhibit the symptoms of another Th2-mediated disease, experimental allergic asthma, confirming the protective role of VDR deficiency in Th2-cell direct diseases^20^.

**Fig. 8:**
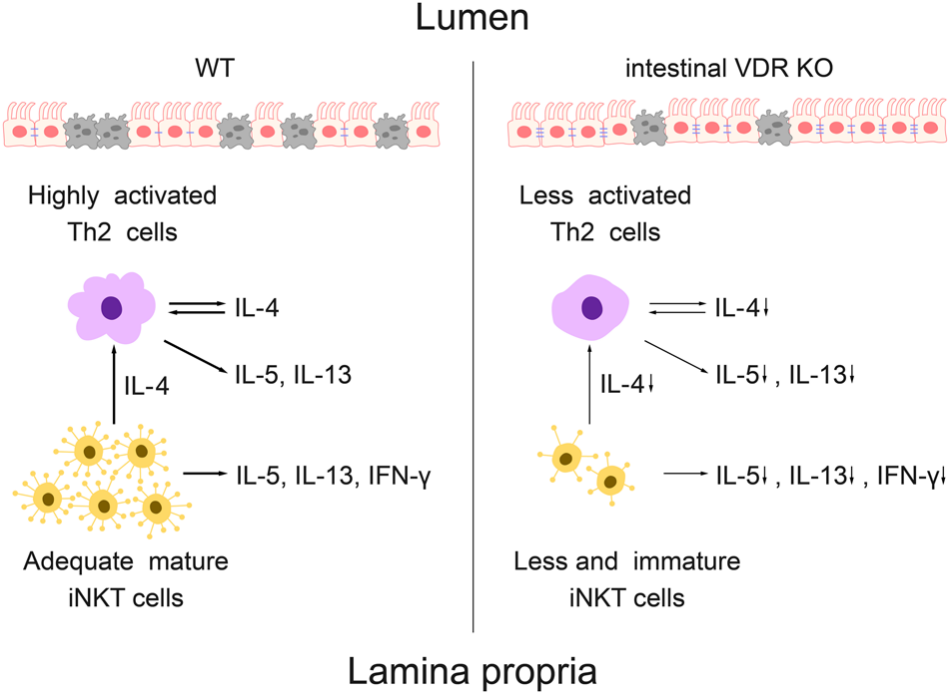
Schematic illustration of how intestinal VDR knockout ameliorate oxazolone-induced colitis. In WT mice, oxazolone stimulation activated both Th2 cells and iNKT cells, resulting in release of IL-4, IL-5, IL-13, and IFN-*γ*. The increase in IL-4 would further activate Th2 cells to produce more cytokines. Those cytokines would aggravate intestinal damage and gut inflammation, resulting in excessive epithelial cell death and barrier destruction. However, when VDR was knocked out, iNKT cells could not proliferate and mature normally. Therefore the production of cytokines would be significantly reduced. Meanwhile, Th2 cells were not as much activated without VDR. Reduction in the release of IL-4 also depressed the activation of Th2 cells. All these contributed to the milder inflammatory response in intestinal VDR knockout mice compared to the wildtype mice.

VDR deficiency not only resulted in Th2 cell dysfunction but also with no less importance, the loss of number and function in iNKT cells. iNKT cells played an important role in tumor surveillance, immune response to infectious pathogens, prevention of autoimmune diseases, and self-tolerance maintenance. They were early precursors of cytokines that shaped the development of T cells^21^. As to the colitic models, NKT cells were protective against DSS-induced colitis but detrimental in oxazolone-induced colitis by producing IL-13^14^. VDR was essential for the development and maturation of iNKT cells, in that VDR^−/−^ iNKT cells would experience increased apoptosis in the early phase and halt in maturation in the late phase that failed to express NK1.1^12^. In this case, much less iNKT cells could be produced and those existed had compromised function, such as decreased production of IL-4 and IFN-*γ*^1^. This would further reduce the activation of Th2 cells. In our study, the dramatic increase in the number of iNKT cells after oxazolone challenge did not happen in VDR^−/−^ mice. This led to inadequate secretion of cytokines that might further stimulate the Th2 cell response. In this case, oxazolone-induced colitis was much less severe than in those WT controls with normal iNKT response (Fig. 8).

Intestinal VDR knockout, however, may cause complicated local immunological changes other than those mentioned above. FoxP3^+^CD4^+^ regulatory T cells were reduced and the production of IL-10 decreased. Meanwhile Th17 cell might be activated and produce more IL-17. This increase of Th17 might have been limited by the deficiency of iNKT cells^22^. Decrease in IL-10 and increased IL-17 might somehow aggravate intestinal inflammation. However, this aggravation had been overwhelmed by the significant reduction in Th2 and iNKT response. There were also some other possible mechanisms, such as the roles of CD8*αα*T cells and the type 2 NKT cells, that required further investigation.

There seemed to be some divergence between our findings and clinical evidence. One study stated that serum 25(OH)D level was negatively associated with disease activity and pro-inflammatory cytokine expression while positively associated with the expression of tight junction proteins^23^. A study that associated prediagnostic serum vitamin D level with IBD found no association between them^2^. One explanation was that lack of VDR and vitamin D deficiency may not have the same influence on a disease. As shown in our study, different serum vitamin D status did not cause any significant change in body weight and survival rate in WT mice. This confirmed that VDR, rather than vitamin D, was the key factor in the regulation of Th2 and iNKT response. This could explain why vitamin D supplementation did not influence the level of IL-4 in UC patients^11^.

Another possible explanation for this was the different source and role of IL-13 between mice and human. In oxazolone-induced murine model, IL-4 was the key mediator and IL-13 was mostly produced by iNKT cells. But in human the key mediator of UC was IL-13 that was not produced by iNKT cells^1^. The divergence between clinical and experimental findings had also been observed in asthma^24,25^, indicating more mechanisms and interactions to be explored in the future.

In conclusion, this study found that intestinal-specific VDR knockout protected against oxazolone-induced colitis in mice. This was achieved by blocking Th2 cell response as well as reducing the number and function of iNKT cells in lamina propria. Our findings may offer some explanation to the controversy on the role of vitamin D in IBD. When confirmed, the precise regulation of VDR might be a novel treatment for IBD.

## Material and methods

### Animals

This study was approved by the Institutional Ethical Committee of Shengjing Hospital, China Medical University. VDR^flox/flox^ mice that carry LoxP sites flanking the VDR gene and villin-CRE mice was purchased from Cyagen Biosciences (Suzhou, China). Their genetic background was C57BL/6J. Intestinal-specific VDR knockout was achieved by crossing VDR^flox/flox^ mice with villin-CRE mice. All the mice were kept in specific pathogen-free static cages with a light/dark circle of 12 h. Chow pellet and tap water was available at libitum. Mice used for experiments were 8–10 weeks old. The control group consisted of wildtype C57BL/6J mice 1:1 matched according to age, gender, and body weight. The female:male ratio was 1:1 in each experiment group.

### Induction of colitis

Mice were anesthetized by injecting a cocktail of xylazine (Rompun 2%; Bayer AG, Leverkusen Germany) and ketamine (Ketavest; 100 mg/ml; Pfizer, NY, USA) intraperitoneally (i.p.). Oxazolone (862207, Sigma-Aldrich, St. Louis, MO, USA) was dissolved in 50% ethanol with the concentration of 5%. Oxazolone was given at a dose of 5 μl/g body weight respectively per rectum with an 18-gauge stainless steel gavage needle. The control group was given the same volume of 50% ethanol without oxazolone.

### Macroscopic and microscopic histological evaluations

The presence or absence of diarrhea was observed and recorded. The disease activity index was calculated for each animal on the basis of stool consistency, rectal bleeding and weight loss percentage^26^. The mice were sacrificed on day 5 after oxazolone injection. When dissecting the colon, the presence or absence of adhesion was noted. The entire colon was harvested, photographed, and observed thoroughly. A 5 cm length of the distal colon was cut open longitudinally, washed with water and extended on a plastic block for the observation of ulceration. The colonic damage score was calculated according to a macroscopic scoring system^27^. A similar section of colon was fixed overnight with 4% formaldehyde in phosphate buffer saline (PBS, pH = 7.4), dehydrated with graded alcohol, placed in xylene and embedded in paraffin. Sections (4 μm) were stained with H&E (Beyotime Institute of Biotechnology, Haimen, China) at room temperature. Five areas were randomly chosen in each section and examined at 200× magnification. In each field, colon microscopic scoring was performed independently by two pathologists who were blinded to the study design according to a microscopic scoring system^28^.

### TUNEL staining

Terminal deoxynucleotidyl transferase-mediated dUTP nick-end labeling (TUNEL) staining was performed on sections of the distal colon to detect intestinal cell apoptosis. An In Situ Cell Death Detection Kit, POD (Roche Diagnostics, Indianapolis, IN, USA) was used according to the manufacturer’s instructions. The apoptotic index was defined as the percentage of TUNEL-positive-cell-containing crypts in 100 randomly chosen crypts in each colon slide.

### Western blotting

The animals were sacrificed 48 h after oxazolone treatment and the distal colonic mucosal lysates were harvested. The lysates were separated by polyacrylamide gel electrophoresis, and the proteins were transferred electrophoretically onto polyvinylidene difluoride membranes (EMD Millipore, Billerica, MA, USA). Then the membranes were incubated with primary antibodies. The following primary antibodies were used in this study: anti-β-actin (A1978, Sigma-Aldrich, St. Louis, MO, USA), anti-zonula occludens-1 (ZO-1, 339100, Invitrogen; Thermo Fisher Scientific, Waltham, MA, USA), anti-occludin (33–1500, Invitrogen), anti-claudin-1 (71–7800, Invitrogen), anti-claudin-2 (32–5600, Invitrogen), anti-T-bet (14–5825–82, Invitrogen), anti-GATA3 (14–9966–82, Invitrogen), anti-RORγt (14–6988–82, Invitrogen), anti-Foxp3 (14–5773–82, Invitrogen), and anti-NK-1.1 (14–5941–82, Invitrogen) antibodies.

### Measurement of intestinal permeability

The mice were fastedbut allowed water for 4 h before gavage. FITC-conjugated 4-kD dextran (FD4) from Sigma (50 mg/ml) was administered via gavage at 4 μl/g body weight through an 18-gauge stainless steel gavage needle. The blood serum was collected 3 h later. Two hundred microliters of the serum per well were added to a 96-well plate; then, the serum concentration of FD4 was measured using a Synergy HT plate reader (BioTek Laboratories, Inc., WA, USA)^29,30^.

### Real-time PCR

The mice were sacrificed two days after oxazolone treatment. A piece of distal colon approximately 1 cm in length was harvest from almost the same segment in all the mice. Colonic mucosa was isolated by careful scraping and RNA was isolated from the colonic mucosa with Trizol (Invitrogen). First-strand cDNAs were synthesized from 2 μg of total RNA in a 20 μl reaction system with M-MLV reverse transcriptase (Invitrogen) and random primers. Real-time PCR was performed using a Bio-RAD IQ5 real-time system and SYBR green PCR Master Mix (Takara Biotechnology Co., Japan). The relative transcription levels of the mRNAs were calculated according to the 2^−ΔΔCt^ formula. β-2 microglobulin (B2M) was used as an internal control. Mouse real-time PCR primers are shown in Supplementary Table 1.

### CD4 immunofluorescent staining

The sections of the colons were incubated with anti-CD4 (1:200 dilution; Santa Cruz Biotechnology, Inc., Dallas, TX, USA) antibodies, followed by secondary antibodies conjugated with AlexaFluor 488 (1:1500 dilution) from Invitrogen. The immunostained antigens were visualized using a Leica DFC425 fluorescence microscope [Leica Microsystems (Schweiz) AG, Heerbrugg, Switzerland].

### Myeloperoxidase (MPO) activity

MPO was expressed in units per milligram of distal colon tissue and analyzed using a MPO assay kit following the manufacturer’s instructions (CytoStore, Alberta, Canada), as previously reported^28^.

### Flow cytometry

Lamina propria cells were isolated from the colon as described previously^13^. In brief, the colons were dissected, cut open longitudinally, and washed in cold phosphate-buffered saline (PBS). The colons were cut into 1.5 cm pieces and washed in PBS containing 1 mM dithiothreitol for 10 min at room temperature on a shaker, followed by two washes with shaking in PBS containing 30 mM EDTA and 10 mM HEPES at 37 °C for 10 min. The tissues were then digested in RPMI 1640 medium containing DNase I (150 μg/ml; Sigma-Aldrich) and collagenase VIII (150 U/ml; Sigma-Aldrich) with 10% fetal bovine serum at 37 °C in a 5% CO_2_ incubator for 1.5 h. Digested cell suspension was filtered with a 70 μm cell strainer and separated by centrifugation on a discontinuous 40/80% Percoll gradient at 2500 rpm for 20 min at room temperature. Cells were collected for flow cytometry analyses. Before cell staining, anti-CD16/32 antibody (eBioscience) was used to block nonspecific binding to Fc receptors. For intracellular staining, cells were fixed and permeabilized using a Mouse Regulatory T-Cell Staining Kit (eBioscience) following the manufacturer’s instruction.

Staining was done in the Staining Buffer (eBioscience) at 4 °C. Fluorescence-activated cell sorting (FACS) was performed in BD LSRFortessa (BD Biosciences) and the data were analyzed with FlowJo software version 7.6.1 (Treestar, Ashland, OR, USA). The peridinin chlorophyll protein-Cy5.5 (PerCP-Cy5.5)-labeled anti-TCRβ antibody (45–5961–82, Invitrogen) and phycoerythin (PE)-labeled *α*-GalCer-loaded CD1d tetramer (ProImmune Inc, Oxford, UK) were used as cell surface markers of iNKT cell subpopulations.

### Vitamin D treatment

Male and female three-week-old C57BL/6J mice were provided by the Center for Experimental Animals of China Medical University. The mice were kept in specific pathogen-free static cages under ultraviolet B-free incandescent light to minimize endogenous vitamin D production. They were randomized into three groups with the gender ratio of 1:1 in each group. A vitamin D deficient diet (Harlan Teklad, Madison, WI, USA) was given to one group, namely the vitamin D deficient (VDD) group. The other two groups were given normal chow pellets and tap water. The vitamin D supplement group, namely the Pari group, were treated with a vitamin D analog paricalcitol (Sigma) dissolved in propylene glycol:ethanol = 90:10 at 0.5 μg/kg body weight while the other two groups were given the dissolvent only. Paricalcitol or vehicle was given through i.p. injection 30 min before, 1 and 3 days after induction of colitis. Oxazolone was given to all of them as previously described.

### Statistical analyses

The continuous data were presented as the mean ± standard deviation (SD). Relative density comparisons were performed with ImageJ software (1.52a; National Institutes of Health, Bethesda, MD, USA). Continuous variables between groups was compared using two-sided Student’s *t*-test or one-way ANOVA (followed by Games-Howell test) with GraphPad Prism software 6.0 (GraphPad Software Inc., La Jolla, CA, USA) and SPSS 23.0 (SPSS Inc., Chicago, IL, USA). Rank data were analyzed with Wilcoxon rank test. Generally *P* < 0.05 was considered significant.

## Supplementary information

Supplementary Table 1
