# Correction: Intestinal vitamin D receptor knockout protects from oxazolone-induced colitis

**DOI:** 10.1038/s41419-020-02913-z

**Published:** 2020-08-21

**Authors:** Yongyan Shi, Ziyun Liu, Xuewei Cui, Qun Zhao, Tianjing Liu

**Affiliations:** 1 Department of Pediatrics, Shengjing Hospital of China Medical University, Shenyang, China; 2 Department of Medicine, Division of Biological Sciences, University of Chicago, Chicago, USA; 3 Department of Pediatric Orthopedics, Shengjing Hospital of China Medical University, Shenyang, China

**Keywords:** Inflammatory bowel disease, Experimental models of disease

Correction to: *Cell Death and Disease*

10.1038/s41419-020-2653-3 published online 15 June 2020

The original version of this Article contained an error in the author affiliations.

The affiliation of Tianjing Liu with Department of Pediatrics, Shengjing Hospital of China Medical University, Shenyang, China was inadvertently omitted.

This has now been corrected in both the PDF and HTML versions.

