# Supplementary material for: Intestinal vitamin D receptor knockout protects from oxazolone-induced colitis
Source: Cell Death Dis. 2020 Jun 15;11(6):461. doi: 10.1038/s41419-020-2653-3 (PMC7296018; doi:10.1038/s41419-020-2653-3)
Supplement: Supplementary file 1 — Supplementary Table 1 [file 41419_2020_2653_MOESM1_ESM.doc]

**Supplementary Table 1 Primer sequences used for real-time PCR.**

| **Primer name** | **Forward (5’ - 3’)** | **Reverse (3’ - 5’)** |
| --- | --- | --- |
| mouse TNF-α | ATGAGCACAGAAAGCATGA | AGTAGACAGAAGAGCGTGGT |
| mouse IFN-γ | TTCTTCAGCAACAGCAAGGC | TCAGCAGCGACTCCTTTTCC |
| mouse IL-4 | AACGAGGTCACAGGAGAAGG | TCTGCAGCTCCATGAGAACA |
| mouse IL-5 | ACCGAGCTCTGTTGACAAG | TCCTCGCCACACTTCTCTTT |
| mouse IL-6 | CCTCTGGTCTTCTGGAGTACC | ACTCCTTCTGTGACTCCAGC |
| mouse IL-10 | ATAACTGCACCCACTTCCCA | GGGCATCACTTCTACCAGGT |
| mouse IL-13 | GCAGCATGGTATGGAGTGTG | TGGCGAAACAGTTGCTTTGT |
| mouse IL-17A | TCTCCACCGCAATGAAGACC | CACACCCACCAGCATCTTCT |
| mouse TGF-β1 | CCTGCAAGACCATCGACATG | TGTTGTACAAAGCGAGCACC |
| mouse T-bet | CATCACTAAGCAAGGACGGC | TATAAGCGGTTCCCTGGCAT |
| mouse GATA-3 | GGCCAGGCAAGATGAGAAAG | AGCTGTACTCGGGCACATAG |
| mouse RORγt | TCCCGAGATGCTGTCAAGTT | GCCCATCTGAGAGCCCTAAA |
| mouse Foxp3 | CCAGCTCTACTCTGCACCTT | GCCTTGCCTTTCTCATCCAG |
| mouse B2M | CGGCCTGTATGCTATCCAGA | GGGTGAATTCAGTGTGAGCC |
